# Diarrheal disease risk in rural Bangladesh decreases as tubewell density increases: a zero-inflated and geographically weighted analysis

**DOI:** 10.1186/1476-072X-10-41

**Published:** 2011-06-15

**Authors:** Margaret Carrel, Veronica Escamilla, Jane Messina, Sophia Giebultowicz, Jennifer Winston, Mohammad Yunus, P Kim Streatfield, Michael Emch

**Affiliations:** 1 Department of Geography, University of Iowa, Iowa City, IA, USA; 2 Department of Geography & Carolina Population Center, University of North Carolina-Chapel Hill, Chapel Hill, NC, USA; 3 International Center for Diarrheal Disease Research, Bangladesh, Dhaka, Bangladesh

## Abstract

**Background:**

This study investigates the impact of tubewell user density on cholera and shigellosis events in Matlab, Bangladesh between 2002 and 2004. Household-level demographic, health, and water infrastructure data were incorporated into a local geographic information systems (GIS) database. Geographically-weighted regression (GWR) models were constructed to identify spatial variation of relationships across the study area. Zero-inflated negative binomial regression models were run to simultaneously measure the likelihood of increased magnitude of disease events and the likelihood of zero cholera or shigellosis events. The aim of this study was to examine the effect of tubewell density on both the occurrence of diarrheal disease and the magnitude of diarrheal disease incidence.

**Results:**

In Matlab, households with greater tubewell density were more likely to report zero cholera or shigellosis events. Results for both cholera and shigellosis GWR models suggest that tubewell density effects are spatially stationary and the use of non-spatial statistical methods is appropriate.

**Conclusions:**

Increasing the amount of drinking water available to households through increased density of tubewells contributed to lower reports of cholera and shigellosis events in rural Bangladesh. Our findings demonstrate the importance of tubewell installation and access to groundwater in reducing diarrheal disease events in the developing world.

## Background

Matlab, Bangladesh is a rural area located approximately 50 kilometers southeast of the capital city, Dhaka. Diarrheal diseases are endemic in Matlab and across Bangladesh. This is due to a number of factors, including natural aquatic environments of diarrheal causative agents, high population density, low socioeconomic status, and limited access to clean water. The installation of tubewells is a primary method for decreasing diarrheal disease incidence, giving Bangladeshis an alternative to drinking contaminated surface water. These tubewells draw water several hundred feet from the underlying aquifer to the surface by hand pumps. A sanitary seal, typically of concrete, ideally prevents seepage of ground water into the tubewell. Despite their sealed nature, tubewells may still harbor microorganisms, such as fecal bacteria, as a result of their proximity to latrines or contaminated surface water bodies [1].

Despite such possible contamination of tubewell water, previous studies of diarrheal disease and drinking water interactions have suggested that it is not simply the use of alternative groundwater sources that provides protection against infection, but also the quantity and accessibility [2-7]. Put simply, the density of a population sharing a tubewell can lower the protective effect conferred by a water supply. Our study examines the potential impacts of tubewell access on two distinct types of diarrheal disease events among Matlab's residents between 2002 and 2004. In doing so, we address two problems commonly encountered and often overlooked in disease analysis: spatial variation in relationships and a small number of reported events. Geographically weighted regression is used to assess potential spatial variability in relationships across the study area and zero-inflated models to control for low disease counts. By utilizing both methods, we are able to ascertain the impact of tubewell access on cholera and shigellosis patterns without the confounding effects of spatial non-stationarity or rare event reporting.

Cholera and shigellosis are two of the most commonly experienced diarrheal diseases in Matlab. Cholera is a disease caused by ingesting a large number of *Vibrio cholerae *bacteria; the infective dose is 10,000 or greater. Shigellosis is caused by ingesting a much smaller dose of *Shigella *bacteria; sometimes as few as ten bacteria are necessary to cause an infection. The primary symptom of cholera is watery diarrhea, while the primary symptom of shigellosis is bloody diarrhea (dysentery). Cholera bacteria naturally occur in the brackish waters of Bangladesh, while *Shigella *bacteria are linked to human waste contamination of water supplies. We hypothesized that, despite these described differences between the two diseases, tubewell user density would be predictive of diarrheal events for both.

## Methods

### DATA

**Figure 1:**
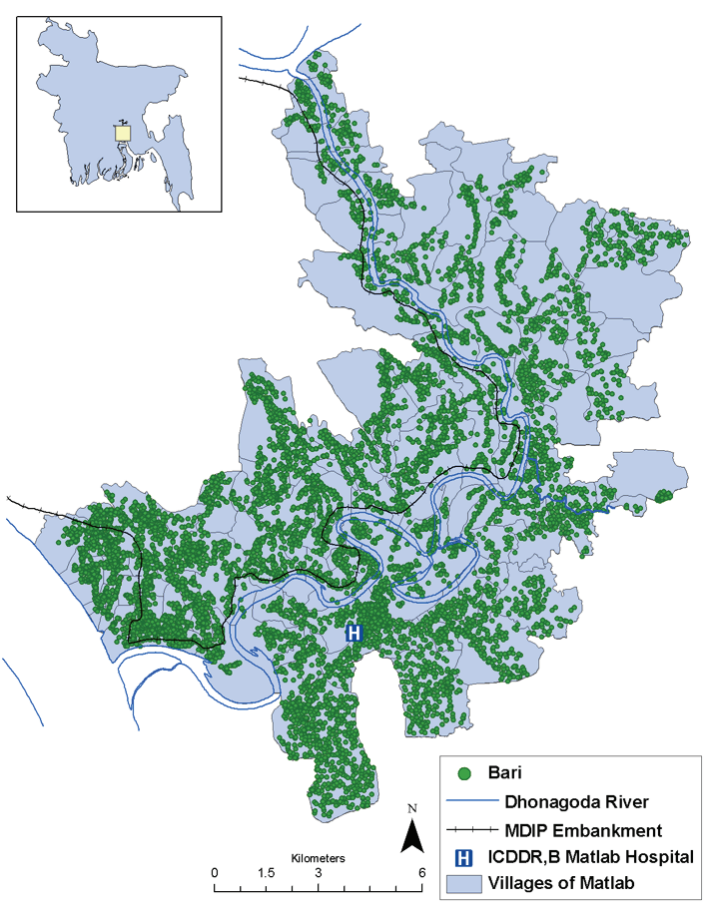
**Location and main geographical features of the Matlab, Bangladesh study area**.

Since 1966, the International Centre for Diarrhoeal Disease Research, Bangladesh (ICDDR,B) has conducted continuous demographic surveillance system (DSS) of Matlab's residents. Each resident is assigned a unique identification number which is used to track their birth, place of residence, migration, health events, marriage, and eventually, death. The 225,000 residents of Matlab live in a network of *baris*, or patrilineally related household clusters, which are then further grouped within 142 villages (Figure 1). Community health workers trained by ICDDR,B visit each *bari *in Matlab once a month and record a variety of demographic and health events which are linked via the identification number to both individual resident's history, the household and *bari *history, and village history.

ICDDR,B operates a hospital in southwest Matlab that specializes in high-quality treatment of diarrheal diseases, respiratory infections, and maternal and child health. Treatment at the ICDDR,B is free for residents of Matlab, and free transportation to the hospital is available for residents who cannot afford travel. The laboratory diagnoses and treatment outcomes of patients are linked to their demographic and health records via the resident's identification number.

The health and demographic surveillance data collected by ICDDR,B is linked to the spatial locations of residence via a Geographic Information System (GIS). The latitude and longitude coordinates collected at the center of each *bari *are geocoded in the GIS, as are the coordinates of other geographic features, such as the ICDDR,B hospital. The location of features in the Matlab GIS are accurate within 10 meters [8]. The spatially-joined environmental and demographic surveillance data allows for the calculation of a variety of measures which are described in further detail below.

**Figure 2:**
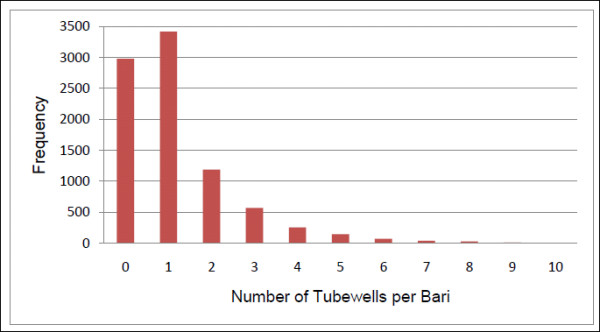
**Distribution of bari tubewell density in Matlab, 2002-2004**. Nineteen baris in Matlab have eleven or more tubewells each, the maximum number of tubewells per bari is 21.

**Figure 3:**
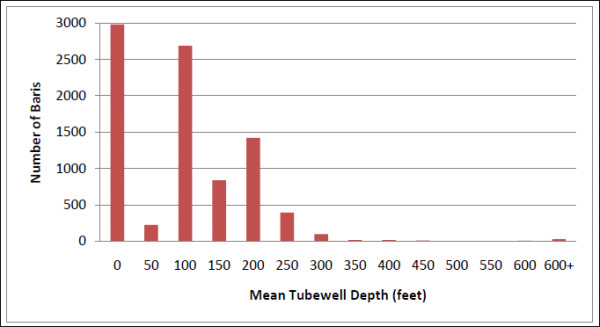
**Distribution of mean tubewell depth in Matlab**. Almost three thousand baris have no private tubewell, coded as zero. The majority of tubewells in Matlab are less than 250 feet deep.

Multiple datasets were joined in the GIS for analysis. The first is a comprehensive global positioning systems (GPS) survey of tubewells conducted in Matlab in 2003 with hand-held receivers used for previous surveys [8]. This survey collected information on tubewell depth and arsenic level for all 10,942 tubewells in the study area. Each tubewell has a unique identification number that links it to a specific *bari *of ownership. For each *bari *we calculated the total number of available tubewells (see Figure 2) as well as an average depth statistic. The average depth statistic was chosen as an effect modifier to control for the possible confounding effects of tubewell depth on tubewell density. Approximately thirty percent of the *baris *in the Matlab study area do not have a privately owned tubewell; their water is drawn from public tubewells or from those belonging to neighboring *baris*. In such cases, their tubewell characteristics were assigned across the board as zero. This retention of *baris *without privately owned tubewells allows us to examine the effect of tubewell density on diarrheal events not only in *baris *with tubewells, but also among those who must either travel to a tubewell. Less convenient access to the main source of drinking water could potentially result in longer storage of drinking water within the home increasing, the likelihood of drinking water contamination if not stored properly, or increased use of surface water for convenience [7]. The distribution of mean tubewell depths in Matlab is seen in Figure 3.

**Figure 4:**
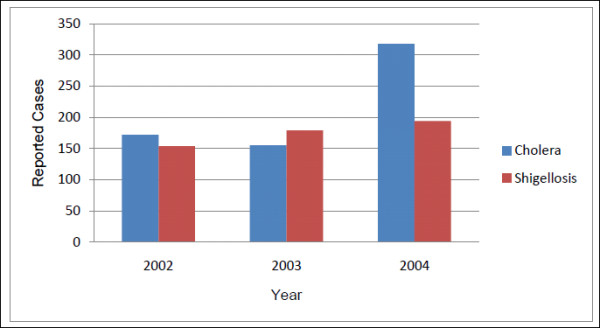
**Annual distribution of cholera and shigellosis, 2002-2004, in Matlab**.

**Figure 5:**
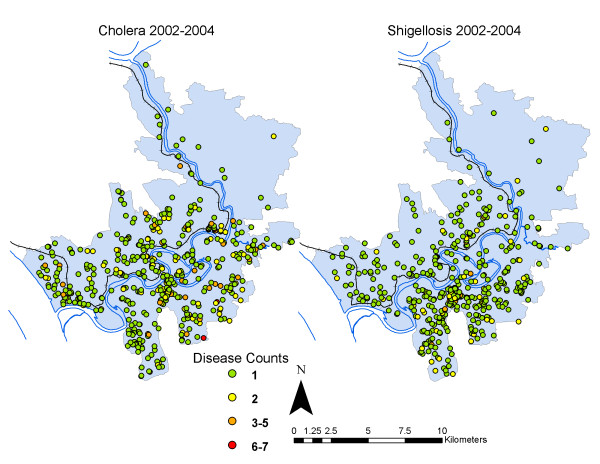
**Spatial distribution of non-zero cholera and shigellosis counts by bari in Matlab, aggregated from 2002-2004**.

All laboratory-confirmed cholera and shigellosis cases reported at the ICDDR,B hospital were aggregated by *bari *for the period January 1, 2002 to December 31, 2004. These years were chosen to extend one year before and one year following the tubewell GPS survey in 2003, to avoid misrepresenting tubewell density prior to the survey, and to avoid confounding by the mass installation of deep tubewells by non-governmental organizations (NGOs) in Matlab in 2005. From 2002 to 2004 there were 645 cholera cases and 527 shigellosis cases in Matlab (annual and spatial distributions are seen in Figures 4 and 5, annual incidence rates in Table 1). The total at-risk population for each *bari *was calculated by summing the mid-year background population counts for 2002 to 2004. Mid-year background populations are derived from the Matlab DSS and account for changes to a *bari's *population due to births, deaths and migrations. Population data gathered by ICDDR,B represents a census of all residents in any given year, and is made possible by the monthly visits of the community health workers to each *bari*.

**Table 1:** Annual incidence rates (per 1000 people) for cholera and shigellosis in Matlab, Bangladesh, 2002-2004

		Year	
**Disease**	2002	2003	2004

Cholera	0.774367	0.691906	1.417605

Shigellosis	0.693328	0.799039	0.864828

The ICDDR,B Matlab hospital is not located in the geographic center of the study area, and varying distance decay effects on patients seeking treatment have been recorded [9-13]. Information regarding residents seeking treatment outside of the study area is currently unavailable. To account for this influence of distance from household to hospital on reporting of cholera and shigellosis, we calculated in the GIS the distance from each *bari *to the ICDDR,B hospital and controlled for it in our models. Straight-line Euclidean distance between *baris *and the hospital was calculated, given that residents of Matlab make use of both road and river networks to travel to the hospital. Because no specific route was known for individual *baris*, a universal distance metric was chosen.

### GWR analysis

Traditional regression models assume that the process accounting for disease incidence is spatially stationary across the study area. This assumption, however, contradicts the goals of spatially-explicit studies whose aim is to understand where and why an outcome differs across a study area. Geographically weighted regression (GWR) models can be used to generate hypotheses about non-stationary relationships as well as pinpoint locations that should be subject to more intensive research [14,15].

GWR produces two types of models: a global regression model that treats relationships as spatially stationary, and local regression models estimated at each observation point. Similar to traditional regression models, GWR estimates a model of global associations with parameter estimates, standard errors and *t*-values. Local regression models are estimated by constructing a spatial weighting matrix and estimating a local weighted regression for each *bari *and its defined neighbors. Observations are weighted based on proximity to each location *i *such that nearer points are assigned greater weight than observations further away, based upon the geographic assumption of spatial autocorrelation. A regression estimate is calculated at each observation, including R^2 ^and other goodness-of-fit measures [15]. A Monte Carlo simulation approach is used to measure significance of local parameters. The variance of observed model parameters is compared against 100 random calibrations of the same model, providing *t*-statistics of significance. The local parameter estimates are specific to each location in both logistic and Gaussian GWR and can be displayed graphically using GIS software, indicating potential spatially-heterogeneous patterns in the relationships between diarrheal disease incidence and variables of interest.

Prior to constructing GWR models, the 8732 *baris *were aggregated into 666 clusters using a 25 × 25 meter grid in ArcGIS software. The grid was constructed to create a smooth, continuous surface where each cell has unique coordinates that can be analyzed using GWR. While each *bari *in Matlab is associated with a specific set of geographic coordinates collected at the center of the *bari*, the coordinates represent points rather than polygons, their dense spatial configuration made conducting GWR without construction of the 25 × 25 meter grid impossible. Model specifications include a logistic distribution in the absence of a negative binomial option to account for low incidence values and overdispersion, and an adaptive spatial kernel function to assign weights for each local regression estimate. The kernel bandwidth was determined by corrected Akaike Information Criterion (AICc) minimization using all data. Adaptive kernel sample size limits were between 166 and 666 *bari *clusters. We selected a Monte Carlo significance testing procedure for the local parameter estimates. The four variables calculated in the GIS were then analyzed against the aggregated counts of cholera and shigellosis cases reported to ICDDR,B: number of tubewells per *bari*, total *bari *population, distance to the Matlab hospital, and the mean tubewell depth per *bari*. Specifically, the aim of constructing geographically weighted regression models was to determine whether tubewell density effects on diarrheal events vary across space, when controlling for other covariates. Local variation would suggest that tubewell access intervention strategies are effective in specific areas within Matlab while a lack of spatial variation would indicate equal effects of tubewell access throughout Matlab.

### ZINB

Many disease datasets contain a large number of zeroes, violating standard regression distribution assumptions and making Poisson or negative binomial regression problematic [16-19]. Zero inflated models (including both zero inflated Poisson (ZIP) and zero inflated negative binomial (ZINB)) account for this overabundance of zero counts and eliminate bias in parameter estimates, and are increasingly used in health studies [11,20,21]. Of the 8732 *baris *in Matlab from 2002 to 2004, 8278 (94.8%) reported zero cholera events and 8308 (95.1%) reported zero shigellosis events. A zero inflated model assumes that these zero counts are made up of both true and false zeroes. In the case of the Matlab dataset, false zeroes would arise from diseased individuals not seeking treatment at the central Matlab ICDDR,B hospital, either because of self-treatment with oral rehydration or other methods, or because medical attention was sought elsewhere in Matlab or the surrounding region. We assume that these false zeroes are greatly outnumbered by true zero counts, however, because of the Matlab ICDDR,B hospital's excellent record in treating diarrheal diseases, with free treatment options and provision of transport to sick individuals.

Zero inflated models are comprised of two components which are analyzed separately. The first component is a count model, containing all positive count observations and a proportion of zero counts that can be normally expected under either a Poisson or negative binomial distribution. The second is a binary model, where zero counts are compared to observations with any positive counts. Zero inflated models thus allow us to estimate the effects of multiple variables on both the likelihood of a *bari *experiencing increased magnitude of disease events (the count model), and the likelihood of a *bari *experiencing no cholera or shigellosis events (the binary model) [18,22,23].

The *pscl *package in R was used for parameter estimation [22,24,25]. A ZINB model was selected due to the overdispersion of aggregated cholera and shigellosis counts with respect to the Poisson distribution. Using the Akaike Information Criterion (AIC) and Log-Likelihood Ratio (LLR) as indications of goodness of fit, the best set of parameters was retained.

## Results

### GWR

Results for the GWR cholera model can be seen in Tables 2 and 3. The global models represent spatially stationary Logistic regression results. Student t-values of ± 1.96 indicate significance at the 0.05 level. Both total *bari *population and average hospital distance are significant predictors of a *bari *cluster reporting a cholera event. The likelihood of a *bari *cluster reporting a cholera event increases as population increases. *Baris *located further from the hospital experience a distance decay effect and are less likely to report cholera. Controls for mean tubewell depth and number of tubewells per *bari *are not significant in the global cholera model.

**Table 2:** Parameter estimates of the cholera geographically weighted global regression model

	Global Model		
	**Estimate**	**Std. Error**	**T value**

Intercept	-0.425	0.301	-1.413

Population (02-04)	0.002	0.000	7.517**

Distance to hospital	0.000	0.000	-9.026***

Number tubewells/*bari*	-0.017	0.012	-1.452

Mean tubewell depth	0.000	0.002	0.002

Significance values: '***' 0.001 '**' 0.01 '*' 0.05

**Table 3:** Comparison of cholera global and local Logistic model diagnostics

	Global Cholera Model	Local Cholera Model
Log Likelihood	-312.10	-292.46

Akaike Information Criterion	635.10	618.41

Corrected AIC	636.09	619.33

**Figure 6:**
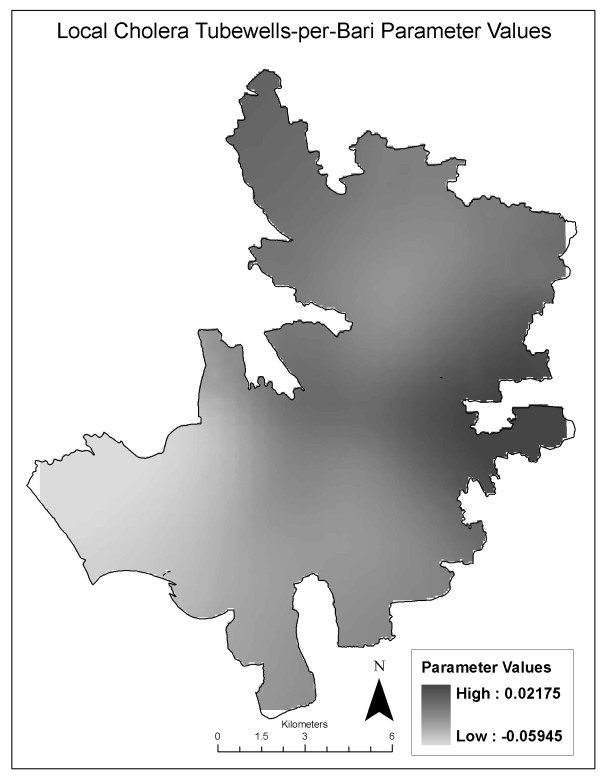
**Local parameter estimates for the number of tubewells per bari variable, cholera model**.

All variables included in the global model were tested in the local cholera model. Decreased AIC values suggest that the local model is a better fit for the data as relationships are non-stationary. A decrease in AIC of 3 points or greater is considered evidence of an improved fit [26]. For the cholera model, the corrected AIC drops 16.76 points, suggesting that the local model is a better fit and spatial heterogeneity may exist (Table 3). However, using the AICc minimization for kernel sample selection resulted in an optimal bandwidth of 366 out of 666 observations. Additional bandwidths with fewer than 366 observations were tested resulting in global models with lower AIC values relative to the local models. Thus, while the drop in the AIC suggests an improved fit in the local model, convergence with more than half of the observations included in each local regression model demonstrates dominant global processes and a lack of spatial heterogeneity. Figure 6 displays the parameter estimates for the primary variable of interest in this study, number of tubewells per *bari*. Parameter estimates are not significant but are shown to demonstrate the limited variability among the relationship between reported cholera and tubewell density across the study area, as indicated by very small range of parameter estimates (between 0.022 and -0.059).

The global GWR results for the shigellosis model are similar to the cholera model results (Table 4). Total *bari *population is a significant positive predictor of reported shigellosis. Average hospital distance is a significant negative predictor of shigellosis, thus the likelihood of a *bari *cluster reporting a case of shigellosis between 2002 to 2004 decreases as distance to hospital increases. Mean tubewell depth and number of tubewells per *bari *variables are not significant predictors in the global shigellosis model.

**Table 4:** Parameter estimates of the shigellosis geographically weighted global regression model

	Global Model		
	**Estimate**	**Std. Error**	**T value**

Intercept	0.270	0.301	0.897

Population (02-04)	0.001	0.000	5.268**

Distance to hospital	0.000	0.000	-10.037***

Number tubewells/*bari*	0.029	0.239	0.121

Mean tubewell depth	-0.003	0.011	-0.301

Significance values: '***' 0.001 '**' 0.01 '*' 0.05

**Figure 7:**
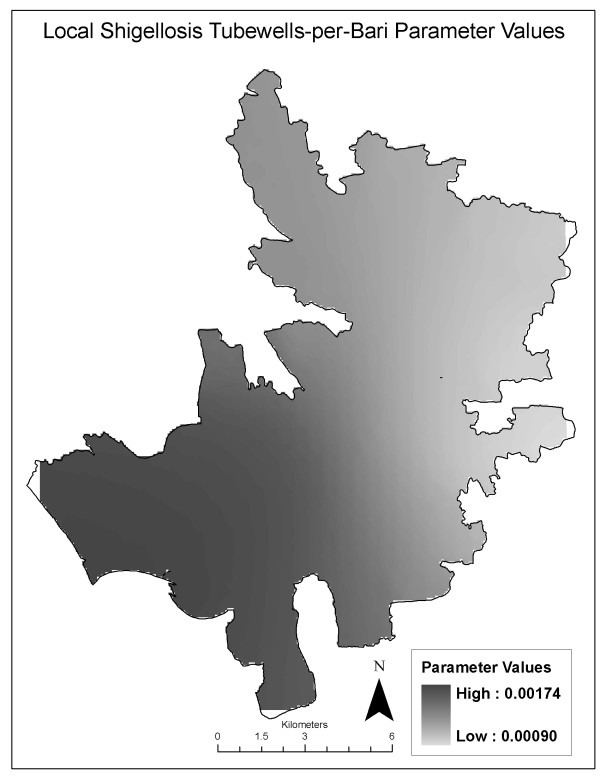
**Local parameter estimates for the number of tubewells per bari variable, shigellosis model**.

The decrease in corrected AIC between the local and global shigellosis models is less than 3 points suggesting that the local model is not a better fit and spatial heterogeneity is not present (Table 5). Using the AICc minimization for kernel sample selection resulted in an optimal bandwidth of 571 out of 666 observations. Additional bandwidths with fewer than 571 observations were tested resulting in global models with lower AIC values relative to the local models. This is evidence of stationary relationships across the study area. Parameter estimates of local relationships between number of tubewells per *bari *and shigellosis are shown in Figure 7 to demonstrate the lack of spatial variability. The lack of variability in Figure 7 is the result of non-significant, uniform local parameter estimates ranging between 0.0009 and 0.0017. GWR results for both the cholera and shigellosis models suggest limited or zero evident spatial heterogeneity in the relationships between event and predictor variables. Therefore, the use of a stationary statistical model that does not account for spatial variation in effects, but does control for low event values and the overabundance of zeros, is appropriate for analyzing these data.

**Table 5:** Comparison of shigellosis global and local Logistic model diagnostics

	Global Shigellosis Model	Local Shigellosis Model
Log Likelihood	-314.07	-308.48

Akaike Information Criterion	638.13	635.58

Corrected AIC	638.23	635.87

### ZINB

Results for the ZINB model for cholera events are presented in Table 6 and the shigellosis model results in Table 7. The expected outcome for zero inflated models is that the direction of association between variables observed in the count model will be reversed in the binary model, such that a positive association with increased counts in the count model will be matched by a negative association with zero events in the binary model.

**Table 6:** Parameter estimates of the cholera zero inflated negative binomial regression model

	Count Model					Binary Model				
	**Estimate**	**Std. Error**	**z value**	**p value**		**Estimate**	**Std. Error**	**z value**	**p value**	

Intercept	-1.7170	0.1641	-10.4620	< 2E-16	***	3.0713	0.4946	6.2100	5.30E-10	***

Number tubewells/bari	-0.0001	0.0350	-0.0030	0.9980		0.6756	0.2192	3.0830	0.0021	**

Population (02-04)	0.0047	0.0008	6.0010	0.0000	***	-0.0792	0.0128	-6.1870	6.12E-10	***

Distance to hospital	-0.1797	0.0204	-8.7900	< 2E-16	***	-0.0037	0.0762	-0.0490	0.9611	

Mean tubewell depth	0.0007	0.0007	1.0190	0.3080		0.0005	0.0019	0.2670	0.7894	

Log (theta)	-1.2490	0.1252	-9.9730	< 2E-16	***					

Significance values: 0 '***' 0.0001 '**' 0.01 '*'

**Table 7:** Parameter estimates of the shigellosis zero inflated negative binomial regression model

	Count Model					Binary Model				
	**Estimate**	**Std. Error**	**z value**	**p value**		**Estimate**	**Std. Error**	**z value**	**p value**	

Intercept	-1.2265	0.2100	-5.8410	5.18E-09	***	1.9978	0.3685	5.4220	5.89E-08	***

Number tubewells/bari	0.0587	0.0381	1.5390	0.1238		0.4105	0.2011	2.0410	0.0412	*

Population (02-04)	0.0020	0.0009	2.3180	0.0204	*	-0.0379	0.0069	-5.5220	3.36E-08	***

Distance to hospital	-0.2631	0.0246	-10.7100	< 2E-16	***	0.0662	0.0606	1.0930	0.2744	

Mean tubewell depth	0.0010	0.0010	1.0390	0.2986		-0.0011	0.0028	-0.3930	0.6946	

Log (theta)	0.4900	0.4080	1.2010	0.2297						

Significance values: 0 '***' 0.0001 '**' 0.01 '*'

For *baris *experiencing cholera in 2002 to 2004, there was a strongly significant (p < .001) relationship to population and hospital distance. *Baris *with larger populations during the time period experienced greater numbers of cholera cases. *Baris *that were far away from the Matlab ICDDR,B hospital experienced lower cholera counts. The number of tubewells that a *bari *had access to was not a statistically significant predictor of cholera counts nor was the mean tubewell depth. The binary model for cholera shows only two statistically significant relationships in the prediction of zero cholera counts: number of tubewells per *bari *and the background population. Increased access to tubewells, as measured by the number of tubewells per *bari*, significantly increases the probability of a *bari *reporting zero cholera events. The background population of a *bari *shows a statistically significant negative relationship with zero counts, such that higher *bari *population decreases the chances of a *bari *reporting no cholera. Mean tubewell depth, as in the count model, showed no statistically significant association in the binary model.

As seen in Table 7, the same predictor variables were statistically significant in the shigellosis count and binary models as the cholera models. In the count model, background *bari *population from 2002-2004 is a significant (p < .001) positive predictor of positive shigellosis counts: as population increases so does the number of shigellosis cases experienced by a *bari*. Distance to the ICDDR,B hospital has the opposite effect on shigellosis case counts: *baris *located further from the hospital are less likely to have high numbers of reported shigellosis cases (p < .001). Neither the number of tubewells in a *bari *nor a *bari*'s mean tubewell depth are significant predictors of shigellosis counts.

Only two variables were significant in the shigellosis binary model: the number of tubewells that a *bari *was able to access and the population of a *bari *from 2002-2004. As the number of tubewells in a *bari *increases, the likelihood of reporting zero shigellosis events increases. As population increases, the reporting of zero shigellosis events decreases at the *bari *level. Distance to the hospital and mean tubewell depth were not significant predictors of whether a *bari *experienced shigellosis during 2002 to 2004.

## Discussion

Geographically weighted regression models were constructed to explore spatial heterogeneity of relationships to determine whether a stationary statistical model was appropriate for the data. Results from both the cholera and shigellosis models suggest that relationships are stationary. The influence of tubewell density on both cholera and shigellosis counts does not vary across the study area suggesting that water access interventions should apply to the whole of Matlab rather than specific target areas.

Results from both the cholera and shigellosis zero inflated models indicate that, when controlling for other population and environment covariates, the number of tubewells that a *bari *is able to access is an important predictor of diarrheal disease events. The models control for background *bari *population size, so regardless of the number of people sharing tubewells, as access to tubewell water increases at the *bari *level the chances of those *baris *reporting no cholera or shigellosis events also increases. This relationship holds true despite the presence of a mean tubewell depth variable in the model, which would control for the effect of varying tubewell depths in some *baris*, or the absence of tubewells completely in one third of the *baris *measured. It is commonly assumed that deeper tubewells draw less polluted water to the surface than shallow tubewells, which may become contaminated as surface water or other material seeps downwards towards the water table [27-29]. While we did not specifically investigate the relationship between shallow tubewells and diarrheal disease events, it was important to account for this potential interaction with the number of tubewells available to a population. Our findings indicate that when the potential for poor water quality in *baris *without tubewells or in *baris *with shallower tubewells is accounted for, the total access to tubewells in a *bari *is a positive predictor for the absence of diarrheal disease.

The distance between a *bari *and the Matlab hospital is a significant variable for both cholera and shigellosis in the count models, wherein *baris *further from the hospital report fewer cases of disease. We interpret these results to mean that there is a distance decay effect in Matlab, such that even in *baris *with disease events, fewer are reported to the hospital. This could be due to the long travel times between *baris *located in the further reaches of the study area, or due to residents seeking medical treatment outside the bounds of the study area. Distance to the hospital is not, however, a significant variable in either of the binary models, suggesting that it is not strongly predictive of whether a *bari *does or does not experience disease. The global GWR models support this assertion.

## Conclusions

Our findings demonstrate the importance of access to clean water in driving diarrheal disease events in the developing world: higher densities of tubewells contribute to lower reports of cholera and shigellosis events. Increasing the amount and quality of clean water available to residents of Matlab and other regions of Bangladesh could lower not only the risk of cholera and shigellosis, but also other diarrheal diseases. This study was limited to the reporting of disease events from 2002 to 2004. Since that time, a large number of very deep tubewells (> 700 feet) have been installed in Matlab, and elsewhere in Bangladesh, by a variety of NGOs to mitigate the impacts of arsenic poisoning from shallow tubewells. Such installation could have dramatic effects on both the distribution and intensity of diarrheal disease events. As part of arsenic mitigation efforts, tubewells were labeled as safe or unsafe for drinking. Residents of *baris *with no safe tubewells have shown a variety of strategies, ranging from travelling to neighboring *baris *or public tubewells to access safe water, switching usage to only one tubewell in a *bari*, or ignoring the warnings and still using arsenic infected tubewells [30]. Our future work will examine the durability of these results in the face of changing water access in Matlab, driven both by individual arsenic mitigation efforts (switching tubewells) and by the large-scale installation of even deeper tubewells by the Bangladeshi government and NGOs.

## Competing interests

The authors declare that they have no competing interests.

## Authors' contributions

MC, VE and ME designed the study, and with JM carried out the statistical analysis and drafted the manuscript. MY and PKS oversaw data design and collected the dataset, and helped revise the manuscript. SG and JW participated in the design of the study and helped to draft the manuscript. All authors read and approved the final manuscript.
